# Does visual feedback during walking result in similar improvements in trunk control for young and older healthy adults?

**DOI:** 10.1186/1743-0003-10-110

**Published:** 2013-11-26

**Authors:** Eric Anson, Russell Rosenberg, Peter Agada, Tim Kiemel, John Jeka

**Affiliations:** 1 Department of Kinesiology, University of Maryland, College Park, MD 20742, USA; 2 Bioengineering Graduate Program, University of Maryland, College Park, MD USA; 3 Neuroscience and Cognitive Science Graduate Program, University of Maryland, College Park, MD, USA; 4 Department of Kinesiology, Temple University, Philadelphia, PA, USA

**Keywords:** Visual feedback, Walking, Balance

## Abstract

**Background:**

Most current applications of visual feedback to improve postural control are limited to a fixed base of support and produce mixed results regarding improved postural control and transfer to functional tasks. Currently there are few options available to provide visual feedback regarding trunk motion while walking. We have developed a low cost platform to provide visual feedback of trunk motion during walking. Here we investigated whether augmented visual position feedback would reduce trunk movement variability in both young and older healthy adults.

**Methods:**

The subjects who participated were 10 young and 10 older adults. Subjects walked on a treadmill under conditions of visual position feedback and no feedback. The visual feedback consisted of anterior-posterior (AP) and medial-lateral (ML) position of the subject’s trunk during treadmill walking. Fourier transforms of the AP and ML trunk kinematics were used to calculate power spectral densities which were integrated as frequency bins “below the gait cycle” and “gait cycle and above” for analysis purposes.

**Results:**

Visual feedback reduced movement power at very low frequencies for lumbar and neck translation but not trunk angle in both age groups. At very low frequencies of body movement, older adults had equivalent levels of movement variability with feedback as young adults without feedback. Lower variability was specific to translational (not angular) trunk movement. Visual feedback did not affect any of the measured lower extremity gait pattern characteristics of either group, suggesting that changes were not invoked by a different gait pattern.

**Conclusions:**

Reduced translational variability while walking on the treadmill reflects more precise control maintaining a central position on the treadmill. Such feedback may provide an important technique to augment rehabilitation to minimize body translation while walking. Individuals with poor balance during walking may benefit from this type of training to enhance path consistency during over-ground locomotion.

## Background

Older adults and some patient populations are at increased risk of falling, with a high probability of those falls resulting in injuries [1,2]. Falls and fall related injuries negatively impact the ability of older individuals to perform daily tasks [3], and substantially impact health care costs. Less easily quantified, but arguably more important, is the reduced quality of life from fall related injuries: disability, dependence on others, lost time from work or household duties [4], and self-restricted social interactions due to fear of falling [5]. It is essential to identify *affordable* solutions to this growing medical, social, and economic problem that are easily accessible to a *large segment of the aging population*. Most falls occur during dynamic activities like walking or transitions from sitting/standing to walking [2,6,7], yet most visual biofeedback for postural control is provided during standing [8-13]. Here we propose a device that has the potential to improve balance through visual feedback of self-motion during walking.

During walking, excessive body/trunk motion has been related to instability in older individuals and individuals with balance disorders [14-16]. Measures of trunk movement during locomotion have been used to identify older individuals with balance problems from individuals without balance problems [17,18]. ML center of mass (COM) displacement during walking increased with age, even when adjusted for stride velocity [19]. Individuals with unilateral and bilateral vestibular loss have demonstrated impaired path consistency for goal directed walking [20,21]. It has recently been suggested that responses of trunk translation through space versus orientation of the trunk to vertical in response to visual stimulation reflect different roles (i.e., navigation versus upright stability) of vision during walking [22]. Such findings suggest that the application of position feedback could reduce COM path deviations in older adults.

Several studies have examined the benefit of visual feedback, usually center of pressure (COP) position feedback during standing, with mixed results regarding improved standing postural control and limited transfer to walking [8-13]. Visual feedback reduced sway in both healthy controls and individuals with Parkinson’s disease [10]. COP visual biofeedback training combined with traditional physical therapy did not enhance the effects of traditional physical therapy for individuals recovering from an acute stroke [23]. Stance symmetry feedback improved standing symmetry, but did not enhance recovery of a symmetrical walking pattern [12]. Visual feedback paradigms emphasizing weight shifting demonstrated more consistent carryover from standing to walking, possibly related to the shared dynamic weight shifting component required for both obstacle avoidance and walking [24]. Balance strategies during walking are not the same as standing [25]; therefore, providing visual feedback during walking (compared to standing) may be more effective for improving balance during walking [26,27].

The use of treadmills in rehabilitation, to normalize a walking pattern is supported by only minor differences in electromyographic, kinematic, and force between over-ground and treadmill walking [28-31]. Despite this, there are only a few reports on the use of augmented visual feedback during treadmill walking; most have not provided visual feedback to improve control of trunk motion, rather the goal was to improve foot placement or improve use of a robotic assistive device for walking [32,33]. Verhoeff et al (2009) provided multisensory (visual, vibratory, and auditory) cues signaling excessive trunk tilt during over-ground walking; however, no directionally specific trunk sway information was provided by the visual cues [34]. Due to the multisensory nature of the feedback in that study, it is unclear the specific role that visual feedback played in reducing trunk motion during walking. A case report described improvement in frontal plane gait mechanics after three weeks of training using real time visual feedback, verbal cues, and virtual reality for an individual with a transfemoral amputation [26]. The expense of the virtual reality systems such as that used in this case study would be prohibitive for most clinics and hospitals, a limitation to its broad application. Moreover, the improvement in frontal plane gait mechanics may not be solely attributable to the visual feedback.

Here we implemented a novel *affordable* approach to determine 1) whether augmented visual position feedback provided during treadmill walking would reduce AP and ML trunk motion variability during walking and 2) age related differences in ability to use feedback. Understanding how visual feedback influences body motion will provide insight regarding rehabilitation options for visual feedback to improve control of body movements during walking.

## Methods

### Subjects

Twenty healthy adults, 8 males and 12 females participated in this study. The participants were grouped by age as younger (mean ± SD 22.6 ± 4.9), and older adults, (mean ± SD 72.6 ± 5.8), participants over age 65 were considered older in this study. All subjects were by self report free from any neurological or recent (prior 12 months) musculoskeletal injury, balance disorder, or vertigo. Young adults were recruited by fliers and word of mouth. Older adults were recruited through an advertisement in a newspaper with a readership age greater than 55 years old. Respondents to the advertisement were screened by phone to verify age and health eligibility before scheduling a participation session. This study was approved by the University of Maryland Institutional Review Board. All subjects provided written informed consent prior to participation.

### Apparatus

#### Virtual reality environment

**Figure 1:**
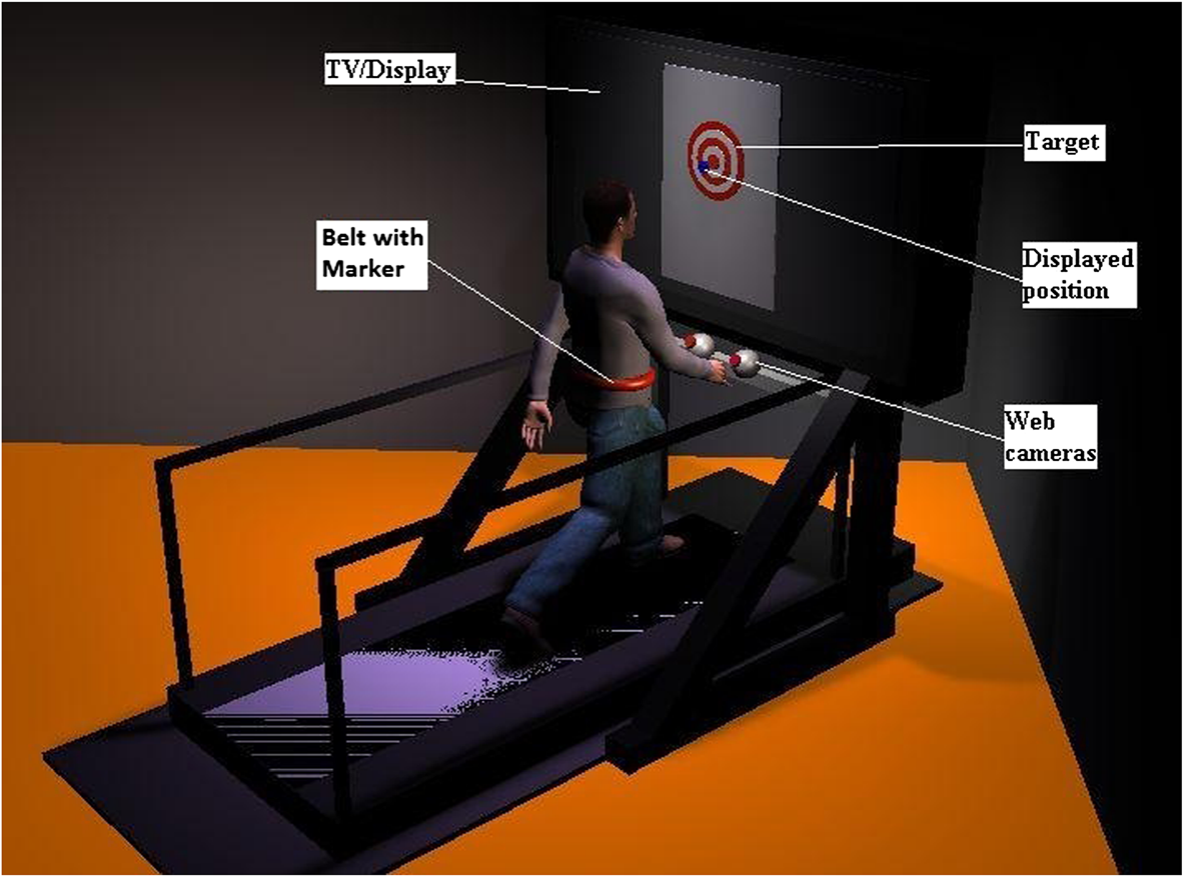
**Illustration of the experimental set-up.** Subjects stood or walked on the treadmill in front of a wide screen TV. A display of their position on the treadmill was indicated by a cursor over a bulls-eye target as a goal area. Depicted is the feedback condition. The TV was turned off and covered with a cloth for the no-feedback condition.

Subjects walked or stood on a treadmill with belt dimensions 0.51 × 1.52 meters (Cybex Trotter 900 T, Cybex International, Inc., USA) approximately 0.6 meters in front of a 1.27 meter wide screen TV (Samsung LN52A550, Samsung, USA) aligned with the front edge of the treadmill belt, shown in Figure 1. The visual display consisted of a grey background textured to look like a treadmill belt with a red and white bull’s-eye target superimposed. This image was presented from a top down (bird’s eye) camera perspective. The diameter of the ten rings of the bull’s-eye increased successively by one inch (total target diameter - 10 inches). The visual display was created using custom scripts in Vizard (WorldViz, USA), on a desktop computer (Dell PWS650 Dell, USA). The position of a colored marker, worn at the height of the navel, was tracked using two webcams (Logitech Orbit AF, Logitech International S.A., USA). Stereoscopic calibration was accomplished using open source code in MATLAB (Mathworks, USA) [35]. The position of the marker was displayed as a cursor on the TV screen. Cursor movement in the vertical direction corresponded to anterior-posterior (AP) movement of the subject on the treadmill, while right-left movement of the cursor corresponded to medio-lateral (ML) movement of the subject on the treadmill. This two dimensional representation of the cursor movement was similar to descriptions of COP feedback displays in previous literature [10-12]. Cursor motion on the screen was scaled relative to the display resulting in a 1:1 ratio of subject motion to cursor motion.

#### Kinematics

**Figure 2:**
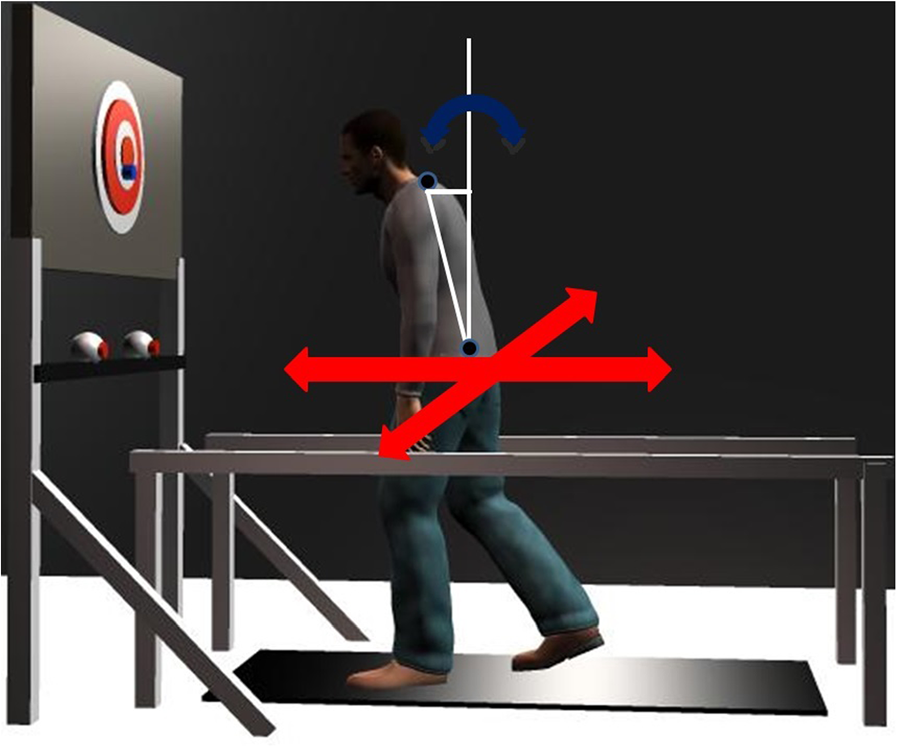
**Illustration of the difference between trunk translation and trunk orientation.** The red arrows depict trunk translation as defined by displacement of the markers on the lumbar vertebrae in AP or ML (neck translation was defined in a similar way). Curved blue arrows represent trunk orientation defined as the AP or ML difference in position of the cervical and lumbar markers, depicted by the horizontal white line.

Kinematics for the young adults were recorded using an Optotrak camera system (Northern Digital Inc., Canada) connected to a desktop computer (Intel Xeon CPU, Dell, USA). Kinematics for the older adults were recorded using a Vicon MX40 (Vicon Motion Systems Inc., USA) camera system connected to a desktop computer (Intel Xeon CPU, Vicon Motion Systems Inc., USA). All kinematics were recorded from the trunk and the right side of the body at a sampling frequency of 120 Hz. Markers were placed at the following anatomical locations: fifth metatarsal, heel, lateral maleolus, lateral femoral condyle, greater trochanter, third lumbar vertebrae, seventh cervical vertebrae, acromion, and head (occiput, left/right temple). Lumbar translation was defined as the AP or ML displacement of the marker on the lumbar vertebrae. Neck translation was defined as the AP or ML displacement of the marker on the cervical vertebrae. Trunk angle (orientation) was defined as the AP or ML difference in position of the cervical and lumbar markers. The difference between cervical and lumbar position in the AP or ML direction is approximately proportional to the trunk angle relative to vertical in the sagittal or frontal plane, respectively, when this angle is small. This allows a direct comparison between measures of trunk orientation and trunk translation using the same units [22]. Trunk translation and orientation are illustrated in Figure 2.

### Procedure

All subjects demonstrated that they were able to walk comfortably at 1.39 m/s (approximately 3.1 miles per hour) without hand rails under conditions of no-feedback and feedback prior to data collection [36,37]. Television height was adjusted for each subject to center the screen at the subject’s approximate eye height. All subjects were able to use their body movement to control cursor movement to the desired location of the bull’s-eye, as the task during feedback conditions was to center the cursor on the bull’s-eye. Subjects were instructed to look straight ahead at the covered TV screen during no-feedback trials. All subjects were given approximately 30 seconds to reach a steady state walking pattern prior to starting each 240 second trial. The experimental design consisted of two different visual feedback conditions: 1) no feedback (NFB) with the TV off and covered; 2) a ten inch diameter bulls-eye target with a cursor to indicate current position (FB). Each condition was presented randomly in five blocks of two trials. Between trials subjects were asked to perform 5 mini-squats to reduce motor memory and were given a standing rest break lasting 1 minute. Between blocks (or as needed to prevent fatigue) all subjects received a seated rest for 3-5 minutes to reduce fatigue.

### Data analysis

#### Power spectral density (PSD)

Fourier transforms of the AP and ML kinematics (lumbar position, neck position, and trunk angle) were calculated. One-sided power spectral densities (PSDs) using Welch’s method with a 20 second Hanning window and one half overlap were then calculated with these transforms [38]. Geometric means of the PSDs were averaged across trials for each subject. For each subject, PSDs were divided into two frequency categories: 1) “below the gait cycle” which included frequencies in the range .05 - .7 Hz; and 2) “gait cycle and above” which included frequencies .75 – 5 Hz. The cut-off frequency of .7 Hz was selected as the upper bound to define “below the gait cycle” as this frequency was below the range of cycle-by-cycle values of gait frequency for all subjects. Frequencies below the gait cycle represent very slow translational or angular oscillations of the body while walking on the treadmill.

#### Motion variability

To evaluate motion variability with visual feedback compared to no feedback, position variance below (.05 - .7 Hz) and above (.75 – 5 Hz) the gait cycle was calculated. Variance for AP and ML trunk kinematics was computed as the integral of the position PSDs using the trapezoid function (trapz.m) in MATLAB (Mathworks, USA). Variance modulated by visual feedback was the difference between variance for no-feedback trials and variance for visual feedback trials.

#### Gait kinematics

Using right leg kinematics, stride time, gait frequency, stride length stance time, stance percentage and their coefficients of variation were calculated. Heel-strike was defined as the local minima of the heel marker in the vertical direction and toe-off was identified from the limb axis minima [32,39,40]. This kinematic method was previously validated against force plate measurements with less than 2% error for detection of heel strike and toe off events [39,40]. The limb axis minima was defined as the local minima of the angle formed by the fifth metatarsal-hip axis in the sagittal plane, with the hip being the origin. Stride time was the average time between successive toe-off events. Stance time was the average time from heel-strike to toe-off. Stride length was computed as the average AP displacement of the heel marker between successive heel-strikes. Coefficients of variation were computed using means and standard deviations for these measures within each trial.

#### Statistical analyses

Statistical analyses were completed using SAS version 9.2 (SAS Institute Inc., Cary, NC). Separately for each group, we analyzed log transformed position variance using a four-way Feedback (FB, NFB) × Direction (AP, ML) × Kinematics (lumbar, neck, trunk) × Frequency (≤ .7 Hz, > .7 Hz) mixed model with all factors repeated, a Kenward-Roger adjustment, and a Tukey-Kramer adjustment for post-hoc within factor comparisons (α = .05). For older adults, this analysis showed a minor increase in variance for only one kinematic variable (trunk angle) in only the ML direction for both feedback conditions in the frequency range including/above the gait cycle (i.e., > 0.7 Hz). For young adults, no significant differences were found between feedback conditions for the high frequency range. Therefore, to better characterize the effects of visual feedback, subsequent analyses were restricted to the low frequency range, below the gait cycle. To determine the effect of age, we analyzed log-transformed position variance using a four-way Age (young, older) × Feedback (FB, NFB) × Direction (AP, ML) × Kinematics (lumbar, neck, trunk) mixed model with Feedback, Direction and Kinematics as repeated factors, an unstructured covariance matrix, a Kenward-Roger adjustment, and a Tukey-Kramer adjustment for post-hoc within factor comparisons (α = .05). Similarly, we analyzed each gait parameter and coefficient of variation using a two way Age (young, older) × Feedback (FB, NFB) mixed model with Feedback as a repeated factor, a Kenward-Roger adjustment, and a Tukey-Kramer post-hoc adjustment (α = .05).

## Results

**Figure 3:**
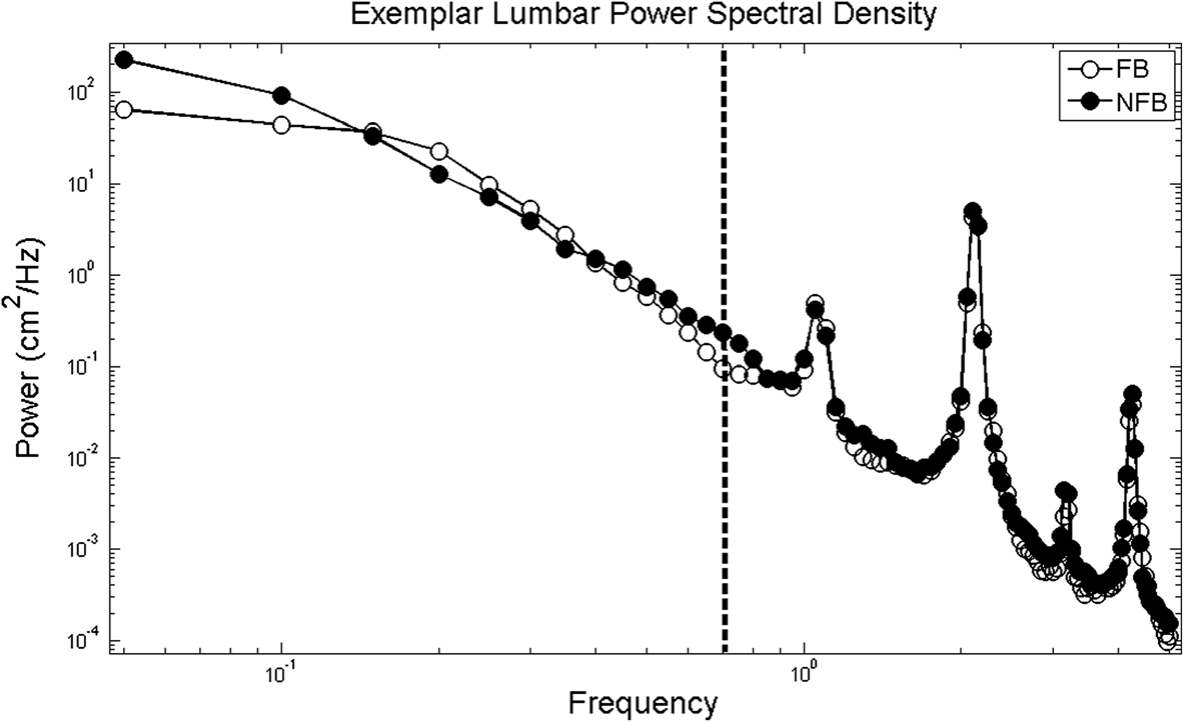
**Exemplar PSD for the AP position of the lumbar marker during a walking trial on the treadmill for one trial for a single subject.** The peak at approximately 1 Hz is the frequency of the gait cycle and the peaks at approximately 2, 3, and 4 Hz are subsequent harmonics. The dashed line marks the frequency (0.7 Hz) separating the frequency bands designated as below the gait cycle and including/above the gait cycle.

Figure 3 shows an exemplar PSD function of the lumbar marker in the FB and NFB conditions for one trial of a single older adult subject. The first peak at approximately 1 Hz represents the average gait cycle frequency and the width of the peak at the base indicates the stride to stride variability of the gait cycle frequency. Subsequent peaks are harmonics of the gait cycle frequency. Differences in spectral power were observed between FB and NFB conditions below the gait cycle frequency (≤ 0.7 Hz), described in detail below. In contrast, spectral power was not significantly different at or above the gait cycle frequency, emphasizing that FB influenced body position only for very slow body movements.

### Kinematic variance

**Figure 4:**
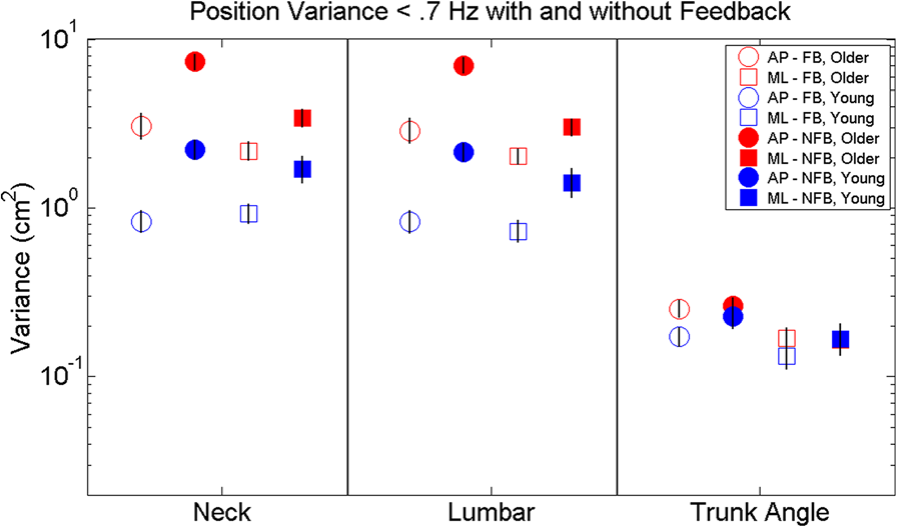
**Position variance.** Open symbols represent the feedback condition and filled symbols represent the no-feedback condition. Red represents older adult and blue represents younger adults. Comparisons between AP (circles) and ML (squares) movement directions for each kinematic segment are presented. Lumbar and neck labels represent translation, while trunk angle represents orientation to vertical. Error bars represent standard error of the mean.

Figure 4 displays position variance up to 7 Hz. The main findings for low frequency position variance were: 1) There was a significant age difference for translation but not orientation responses regardless of visual FB condition, supported by an interaction between age and kinematics (p < .0001); 2) The older adults had significantly greater variance in both the AP and ML directions regardless of FB condition, supported by an interaction between age and response direction (p < .05); 3) Visual feedback significantly reduced movement variance regardless of age for translation, but not orientation, supported by a significant interaction between Kinematics and FB (p < .0001), with no significant three- or four-way interactions including age; 4) Visual FB reduced AP position variance significantly more than ML position variance regardless of age, supported by an interaction between Direction and Feedback (p < .01).

Overall, responses of young adults to visual FB differed from that of older adults in the following ways: 1) Visual FB reduced trunk angle variance from NFB to FB for young adults (*p* < .05), only in the AP direction.

#### General gait measures

Neither older nor younger adults displayed within age group differences across FB conditions for any gait parameters after post-hoc Bonferoni corrections for multiple tests. Older adults demonstrated significantly higher gait frequency (p < .05) compared to the young adults. Older adults also demonstrated significantly higher variability in stride time, stance time, and percentage of time in stance than younger adults (p < .05). Average gait parameters are presented by age and FB condition in Table 1.

**Table 1:** General gait parameters for feedback and no-feedback conditions

	**Older adults**		**Young adults**	
**Gait parameter**	**FB**	**NFB**	**NFB**	**FB**
Stride time (s)*	0.96	0.98	1.05	1.03
C_V_*	.02	.01	.01	.01
Gait frequency (Hz)*	1.05	1.02	.956	.973
Stride length (m)	1.35	1.39	1.42	1.39
C_V_	.02	.02	.01	.01
Stance time (s)	0.62	0.63	.654	.642
C_V_*	.02	.02	.01	.01
Stance (%)	64.3	64.4	62.3	62.2
C_V_*	.02	.01	.01	.01

C_V_ is the coefficient of variation for the corresponding gait parameter. Significant differences between age groups are indicated by an *(p < .05).

## Discussion

The novel approach in this experiment demonstrated that concurrent augmented visual position FB provided during treadmill walking minimized trunk translation. Reduced trunk translation was specific to low frequencies of trunk movement, with translational (not angular) movements, but without changing the characteristics of gait. Our results have implications as a potential rehabilitation method for those with impaired control of trunk movement during locomotion.

The primary reduction in trunk movement variance was observed at low frequencies of body movement, well below the frequency of the gait cycle (≈ 1 Hz). A number of factors favor such low frequency adjustments. First, frequencies of body movement up to .7 Hz contain significant power during standing posture and locomotion (during locomotion the percentage of total power was 38% for trunk angle and 77% for trunk translation). These relatively large, slow movements of the body are amenable to visual control: they are easier to detect visually than smaller movements, vision is known to have slower processing loops than modalities like proprioception [41], and vision is known to influence low frequency movement during standing [42]. Furthermore, the visual feedback presented here required voluntary adjustments, which necessitates slower processing than reflexive adjustments.

Reduction in very low frequency translational movements while walking on the treadmill minimized trunk translation while walking on the treadmill. The functional correlate of reduced body translation during walking is enhanced path consistency. Clinical tests for dynamic walking balance include assessments of path deviation, a difficult task for individuals with impaired balance [43]. The older adults were able to reduce their low frequency trunk translation variability during walking with visual FB, displaying similar or lower variability than young adults without visual FB. The lack of significant change in the ML direction for the young adults with respect to neck translation is likely due to the lower variance for neck ML translation compared to that of older adults. This is consistent with previous reports that older adults present with increased COM displacement in the ML direction while walking [17-19]. The reduction in ML COM translation variability suggests a specific rehabilitation avenue for older individuals and individuals with balance disorders to improve control of body movement during walking. The reduction in ML COM translation variability may be interpreted as an increase in path consistency.

There was a significant reduction in low frequency AP translation variability of the lumbar and neck regardless of age. This corresponds to less drift in the AP direction while walking on a treadmill. The functional relevance of this is unclear as the implicit task for treadmill walking is to not “walk off” [44], which can be accomplished in multiple locations on the treadmill. This illustrates a primary difference between using AP COM translation during treadmill and over-ground walking. AP COM translation during over-ground walking defines the forward path, but on a treadmill is only task relevant at the extreme edges. Reduction in movement variance from visual FB was found for trunk translation in both young and older adults. In contrast, the young adults showed a small but significant reduction in low frequency AP trunk orientation movements, with no effect observed for trunk angle in older adults (see Figure 4). The response specificity observed for older adults to position visual FB during walking may have implications for rehabilitation. In response to multi-modal biofeedback of their trunk angle sway older adults were able to reduce ML trunk angle sway during walking [45]. Providing visual feedback specifically related to the rehabilitation movement goals (i.e. trunk translation versus trunk orientation) may result in greater benefit and functional carryover.

Visual FB presented in this way may be able to reduce age or pathology associated increases in translation of the COM during walking [18,19,46]. The translation-specific response to augmented visual FB seen in older adults in this study may provide some insight regarding the mixed effects to visual feedback previously reported [8,11,47]. During walking, translation and orientation of the trunk serve the roles of navigation and upright stability, respectively. Since navigation is not relevant for standing, such separation of function does not apply to standing sway [22]. Thus, it may be inappropriate to provide COM translation FB if the goal is to reduce trunk deviations from vertical while walking. Visual feedback training for path consistency during walking may be more amenable to COM translation FB training.

Finally, an argument could be made that the presence of visual feedback induced a change in control of walking as there is significant literature reporting the influence of vision on gait [48,49]. The lack of difference on the measured gait parameters between feedback conditions demonstrates that average spatial/temporal aspects of walking were unchanged, despite reduced AP and ML trunk translation in space. This is consistent with the idea that cyclic behavior of the legs, path consistency and upright orientation are separate tasks during walking [7,27,50], and demonstrates that path consistency can be modified independent of changes to the average walking pattern. The implication for rehabilitation is that isolated functional impairments in COM translation control may be effectively improved during walking using concurrent visual FB.

This study demonstrated that healthy young and older adults were able to effectively use visual feedback to reduce low frequency trunk translation while walking on a treadmill. A potential advantage of visual feedback provided during treadmill walking versus standing is the more dynamic component of the walking activity. The current results provide proof of concept for a low cost device that provides visual position feedback during walking to minimize excessive body movements. Whether this method of training will improve over-ground walking remains to be seen and is currently under investigation. Such low frequencies of body sway contain the majority of spectral power for standing posture, suggesting that the changes observed may be related to the control of balance during walking. The response-specific nature of this visual feedback may also enable greater carryover to functional mobility. Further research in this area is needed to determine whether other aspects of body movement during walking can be influenced with different types of feedback and to determine whether beneficial carry over effects exist for over-ground walking.

## Conclusion

Visual position feedback provided during treadmill walking minimized trunk motion specific to the nature of the feedback. The response specific effect of the visual feedback indicates that for healthy adults the different aspects of body control during walking (trunk translation vs. trunk orientation) [22], do not respond similarly to the same visual feedback. This suggests that just as different mechanisms are responsible for control of standing and walking balance [7], different mechanisms also underlie control of the upright orientation versus translation of the body during walking. Rehabilitation of balance during locomotion may benefit from provision of specific sensory feedback tailored to these mechanisms.

## Abbreviations

COP: Center of pressure; COM: Center of mass; AP: Anterior-posterior; ML: Medio-lateral; PSD: Power spectral density; Hz: Hertz; FB: Feedback; NFB: No feedback.

## Competing interest

J Jeka, E Anson, and P Agada disclose that they are listed as inventors of the sensory treadmill described in this experiment and a patent application for this device has been submitted.

## Authors’ contribution

EA participated in the design of the study, conducted the data collection with older adults, performed the statistical analysis, and helped draft the manuscript. RR conducted the data collection with younger adults and helped draft the manuscript. PA participated in the design of the study and development of software algorithms. TK participated in the design of the study, helped draft the manuscript, and performed the statistical analysis. JJ conceived of the study, participated in its design and coordination, and helped draft the manuscript. All authors read and approved the final manuscript

## References

[B1] Kannus P Parkkari J Koskinen S Niemi S Palvanen M Järvinen M Vuori I Fall-induced injuries and deaths among older adults JAMA 1999 281 1895 1899 10349892 10.1001/jama.281.20.1895

[B2] Lord SR Ward JA Williams P Anstey KJ An epidemiological study of falls in older community-dwelling women: the randwick falls and fractures study Aust J Public Health 1993 17 3 240 245 8286498 10.1111/j.1753-6405.1993.tb00143.x

[B3] Fuller GF Falls in the elderly Am Fam Physician 2000 61 7 2159 2168 10779256

[B4] Zijlstra GA van Haastregt JC van Eijk JT van Rossum E Stalenhoef PA Kempen GI Prevalence and correlates of fear of falling, and associated avoidance of activity in the general population of community-living older people Age Ageing 2007 36 304 309 17379605 10.1093/ageing/afm021

[B5] Arfken CL Lach HW Birge SJ Miller JP The prevalence and correlates of fear of falling in elderly persons living in the community Am J Public Health 1994 84 565 570 8154557 10.2105/ajph.84.4.565 PMC1614787

[B6] Gabell A Simons MA Nayak US Falls in the healthy elderly: predisposing causes Ergonomics 1985 28 7 965 975 4043031 10.1080/00140138508963219

[B7] Winter DA Human balance and postural control during standing and walking Gait Posture 1995 3 193 214

[B8] Van Peppen RP Kortsmit M Lindeman E Kwakkel G Effects of visual feedback therapy on postural control in bilateral standing after stroke: a systematic review J Rehabil Med 2006 38 3 9 16548079 10.1080/16501970500344902

[B9] Cheng PT Wang CM Chung CY Chen CL Effects of visual feedback rhythmic weight-shift training on hemiplegic stroke patients Clin Rehabil 2004 18 747 753 15573830 10.1191/0269215504cr778oa

[B10] Waterston JA Hawken MB Tanyeri S Jantti P Kennard C Influence of sensory manipulation on postural control in Parkinson’s disease J Neurol Neurosurg Psychiatry 1993 56 1276 1281 8270927 10.1136/jnnp.56.12.1276 PMC1015374

[B11] Sihvonen SE Sipilä S Era PA Changes in postural balance in frail elderly women during a 4-week visual feedback training: a randomized controlled trial Gerontology 2004 50 87 95 14963375 10.1159/000075559

[B12] Winstein CJ Gardner ER McNeal DR Barto PS Nicholson DE Standing balance training: Effect on balance and locomotion in hemiparetic adults Arch Phys Med Rehabil 1989 70 755 762 2802955

[B13] Zijlstra A Mancini M Chiari L Zijlstra W Biofeedback for training balance and mobility tasks in older populations: a systematic review J Neuroeng Rehabil 2010 7 58 21143921 10.1186/1743-0003-7-58 PMC3019192

[B14] Simoneau M Teasdale N Bourdin C Bard C Fleury M Nougier V Aging and postural control: postural perturbations caused by changing the visual anchor J Am Geriatr Soc 1999 47 2 235 240 9988297 10.1111/j.1532-5415.1999.tb04584.x

[B15] Allum JH Adkin AL Carpenter MG Held-Ziolkowska M Honegger F Piechala K Trunk sway measures of postural stability during clinical balance tests: effects of a unilateral vestibular deficit Gait Posture 2001 14 3 227 237 11600326 10.1016/s0966-6362(01)00132-1

[B16] Gill J Allum JH Carpenter MG Held-Ziolkowska M Adkin AL Honegger F Piechala K Trunk sway measures of postural stability during clinical balance tests: effects of age J Gerontol A Biol Sci Med Sci 2001 56 7 M438 M447 11445603 10.1093/gerona/56.7.m438

[B17] Yack HJ Berger RC Dynamic stability in the elderly: identifying a possible measure J Gerontol 1993 48 5 M225 M230 8366265 10.1093/geronj/48.5.m225

[B18] Chou LS Kaufman KR Hanh ME Brey RH Mediolateral motion of the center of mass during obstacle crossing distinguishes elderly individuals with imbalance Gait Posture 2003 18 125 133 14667945 10.1016/s0966-6362(02)00067-x

[B19] Schrager MA Kelly VE Price R Ferrucci L Shumway-Cook A The effects of age on medio-lateral stability during normal and narrow base walking Gait Posture 2008 28 466 471 18400500 10.1016/j.gaitpost.2008.02.009 PMC2583141

[B20] Borel L Harlay F Lopez C Magnan J Chays A Lacour M Walking performance of vestibular-defective patients before and after unilateral vestibular neurotomy Behav Brain Res 2004 150 191 200 15033292 10.1016/S0166-4328(03)00257-2

[B21] Glasauer S Amorim MA Vitte E Berthoz A Goal-directed linear locomotion in normal and labyrinthine-defective subjects Exp Brain Res 1994 98 323 335 8050517 10.1007/BF00228420

[B22] Logan D Kiemel T Dominici N Cappellini G Ivanenko YP Lacquaniti F Jeka JJ The many roles of vision during walking Exp Brain Res 2010 206 3 337 350 20852990 10.1007/s00221-010-2414-0

[B23] Walker C Brouwer BJ Culham EG Use of visual feedback in retraining balance following acute stroke Phys Ther 2000 80 886 895 10960936

[B24] Hatzitaki V Voudouris D Nikodelis T Amiridis IG Visual feedback training improves postural adjustments associated with moving obstacle avoidance in elderly women Gait Posture 2009 29 296 299 18996012 10.1016/j.gaitpost.2008.09.011

[B25] Shkuratova N Morris ME Huxham F Effects of age on balance control during walking Arch Phys Med Rehabil 2004 85 582 588 15083433 10.1016/j.apmr.2003.06.021

[B26] Darter BJ Wilken JM Gait training with virtual reality-based real time feedback: Improving gait performance following transfemoral amputation Phys Ther 2011 91 9 1385 1394 21757579 10.2522/ptj.20100360

[B27] Wollacott MH Bles W, Brandt T Gait and postural control in the aging adult Disorders of posture and gait 1986 Amsterdam: Elsevier 325 336

[B28] Goldberg EJ Kautz SA Neptune RR Can treadmill walking be used to assess propulsion generation? J Biomech 2008 41 1805 1808 18436229 10.1016/j.jbiomech.2008.03.009 PMC2413053

[B29] Lee SJ Hidler J Biomechanics of overground vs. treadmill walking in healthy individuals J Appl Physiol 2008 104 747 755 18048582 10.1152/japplphysiol.01380.2006

[B30] Riley PO Paolini G Della Croce U Paylo KW Kerrigan DC A kinematic and kinetic comparison of overground and treadmill walking in healthy subjects Gait Posture 2007 26 17 24 16905322 10.1016/j.gaitpost.2006.07.003

[B31] Watt JR Franz JR Jackson K Dicharry J Riley PO Kerrigan DC A three-dimensional kinematic and kinetic comparison of overground and treadmill walking in healthy elderly subjects Clin Biomech 2010 25 444 449 10.1016/j.clinbiomech.2009.09.002 20347194

[B32] Banz R Bolliger M Colombo G Dietz V Lunenburger L Computerized visual feedback: an adjunct to robotic-assisted gait training Phys Ther 2008 88 1135 1145 18772279 10.2522/ptj.20070203

[B33] Dingwell JB Davis BL A rehabilitation treadmill with software for providing real-time gait analysis and visual feedback J Biomech Eng 1996 118 253 255 8738792 10.1115/1.2795968

[B34] Verhoeff LL Horlings CG Janssen LJ Bridenbaugh SA Allum JH Effects of biofeedback on trunk sway during dual tasking in the healthy young and elderly Gait Posture 2009 30 76 81 19356934 10.1016/j.gaitpost.2009.03.002

[B35] Bouguet J-Y Camera calibration toolbox for matlab[http://www.vision.caltech.edu/bouguetj/calib_doc/index.html] accessed 2009

[B36] Browning RC Baker EA Herron JA Kram R Effects of obesity and sex on the energetic cost and preferred speed of walking J Appl Physiol 2006 100 390 398 16210434 10.1152/japplphysiol.00767.2005

[B37] Malatesta D Simar D Dauvilliers Y Candau R Borrani F Prefault C Caillaud C Energy cost of walking and gait instability in healthy 65- and 80-yr-olds J Appl Physiol 2003 95 2248 2256 12882986 10.1152/japplphysiol.01106.2002

[B38] Bendat JS Piersol AG Random data: analysis and measurement procedures 2000 3 New York: Wiley

[B39] Borghese NA Bianchi L Lacquaniti F Kinematic determinants of human locomotion J Physiol 1996 494 863 879 8865081 10.1113/jphysiol.1996.sp021539 PMC1160684

[B40] Ivanenko YP Poppele RE Lacquaniti F Five basic muscle activation patterns account for muscle activity during human locomotion J Physiol 2004 556 267 282 14724214 10.1113/jphysiol.2003.057174 PMC1664897

[B41] Fitzpatrick R McCloskey DI Proprioceptive, visual and vestibular thresholds for the perception of sway during standing in humans JPhysiol 1994 478 1 173 186 7965833 10.1113/jphysiol.1994.sp020240 PMC1155655

[B42] Diener HC Dichgans J Guschlbauer B Bacher M Role of visual and static vestibular influences on dynamic posture control Hum Neurobiol 1986 5 2 105 113 3488305

[B43] Shumway-Cook A Woollacott MH Motor control: Theory and practical applications 2001 Baltimore: Williams and Wilkins

[B44] Dingwell JB John J Cusumano JP Do humans optimally exploit redundancy to control step variability in walking? PLoS Comput Biol 2010 6 7 e1000856 20657664 10.1371/journal.pcbi.1000856 PMC2904769

[B45] Davis JR Carpenter MG Tschanz R Meyes S Debrunner D Burger J Allum JH Trunk sway reductions in young and older adults using multi-modal biofeedback Gait Posture 2010 31 465 472 20206528 10.1016/j.gaitpost.2010.02.002

[B46] Swinnen E Baeyens J-P Pintens S Buyl R Goosens M Meeusen R Kerckhofs E Walking more slowly than with normal velocity: The influence on trunk and pelvis kinematics in young and old healthy persons Clin Biomech 2013 http://dx.doi.org/10.1016/j.clinbiomech.2013.06.013 10.1016/j.clinbiomech.2013.06.013 23856336

[B47] Dault MC de Haart M Geurts AC Arts IM Nienhuis B Effects of visual center of pressure feedback on postural control in young and elderly healthy adults and in stroke patients Hum Mov Sci 2003 22 221 236 12967755 10.1016/s0167-9457(03)00034-4

[B48] Konczak J Effects of optic flow on the kinematics of human gait: a comparison of young and older adults J Mot Behav 1994 26 3 225 236 15757838 10.1080/00222895.1994.9941678

[B49] Lamontagne A Fung J McFayden BJ Faubert J Modulation of walking speed by changing optic flow in persons with stroke J Neuroeng Rehabil 2007 4 22 17594501 10.1186/1743-0003-4-22 PMC1913055

[B50] Liang JN Brown DA Impaired foot-force direction regulation during postural loaded locomotion in individuals poststroke J Neurophysiol 2013 110 378 386 23615554 10.1152/jn.00005.2013

